# Prevalence of Type II Diabetes Mellitus Among Adult Population in Medical Department of A Tertiary Care Centre

**DOI:** 10.31729/jnma.5647

**Published:** 2020-12-31

**Authors:** Lochan Karki, Krishna Rana, Manisha Shahi, Anjila Pradhan, Roshina Thapa, Prajwala Yogi, Aliska Niroula

**Affiliations:** 1 Department of Medicine, Himal Hospital, Kathmandu, Nepal; 2 Deurali Primary Health Centre, Nuwakot, Nepal; 3 Himal Hospital, Kathmandu, Nepal; 4 Tribhuwan University Teaching Hospital, Maharajgunj, Kathmandu, Nepal; 5 Kathmandu Medical College-Teaching Hospital, Sinamangal, Kathmandu, Nepal

**Keywords:** *lifestyle behaviours*, *metabolic syndrome*, *Nepal*, *Type II Diabetes Mellitus*

## Abstract

**Introduction:**

Diabetes mellitus is a chronic metabolic disease which is mainly associated with a number of lifestyle behaviours. There is high discrepancy among urban and rural populations with prevalence of diabetes in rural areas as 2.5% and high prevalence of 14.6% in urban areas. Type 2 diabetes mellitus accounts for the majority of all diabetes cases with a number of chronic effects that includes cardiovascular disease, kidney disease, blindness, and disability. This study is done to determine the prevalence of Type II Diabetes Mellitus among adult population in a medical department of a tertiary care centre.

**Methods:**

A descriptive cross-sectional study was done in a medical department of a tertiary care centre of Himal Hospital Private Limited from March to April 2020. Ethical approval was taken from Ethical Review Board of NHRC (Reference Number 752). All the data of the last two years from medical record section were included in the study. The convenience sampling technique was followed. Descriptive statistical analysis was done.

**Results:**

The study showed the prevalence of Type II Diabetes Mellitus among adult population to be 46 (23.93%) (0.23) (C.I= 0.20-0.26).

**Conclusions:**

The prevalence of Type II Diabetes Mellitus was found to be higher than the previous study done in similar settings.

## INTRODUCTION

Diabetes mellitus (DM), the most prevalent non-communicable and common chronic endocrine illnes is mainly associated with a number of lifestyle behaviors, including daily smoking, heavy alcohol drinking, obesity, and reduced physical activity.^1^ Diabetes is accompanied by a marked reduction in patient's quality of life (QOL) and leads to higher disability-adjusted life years than most diseases. Nepal is currently suffering from a double burden of communicable and non-communicable diseases.^2^

According to an estimation done by the World Health Organization (WHO), the cases of diabetes in Nepal are expected to rise from the 436,000 (2% prevalence) in 2000 to 1,328,000 (10% prevalence) in 2030.^3^ Type 2 diabetes mellitus (T2DM) has a number of chronic effects that includes cardiovascular disease, kidney disease, blindness, and disability.^1^ As the number of studies have shown that the burden of non-communicable disease has been on rise especially in urban areas, so this study is intended to find the burden of diabetes in a tertiary hospital of a metropolitan city.^4–5^

This study aims to determine the prevalence of Type II Diabetes Mellitus among adult population of a medical department in a tertiary care centre.

## METHODS

A descriptive cross-sectional study was conducted in Medicine department of Himal Hospital Private Limited. After taking the ethical clearance from ethical review board NHRC (Reference Number 752), the data was collected retrospectively from the medical record section from March to April 2020. The sample size of this study is calculated using the formula,

$$\begin{array}{ll} \text{Sample size }(\text{n)} & \text{= }{\text{Z}}^{\text{2}}\times \text{pq}/{\text{e}}^{\text{2}}\\ & \text{= }{\left(1.96\right)}^{\text{2}}\times 0.06\times (1-0.06)/{(\text{0.05)}}^{\text{2}}\\ & \text{= }87 \end{array}$$

Where,

Confidence Interval (CI) = 95%
Margin of error (e) = 5%
p = prevalence which is taken as 6%^6^
q = (1-p)

Therefore, the calculated sample size was 87. Adding the 10% non-response rate, the sample size that will be taken would be 96.

Convenient (non-probability) sampling technique was applied. Therefore, the sample size was doubled to increase the scientific validity to 192. Data of Patient attending Medicine ward, all patients aged above 18 years were included in the study, whereas patients aged below 18 year, incomplete medical record and the record of other departments were excluded. Permission from the hospital administration was taken. Then specifically designed medical record proforma form was filled. The following bias could happen such as information bias due to missing and repetition of the data.

Data collected via self-administered questionnaire was kept in Microsoft Excel and then edited and checked. After that the data was put in SPSS, the descriptive statistical analysis was done. Frequency, percentages was calculated for binary data and mean and standard deviation were calculated for continuous data after the normality of the data has been checked.

## RESULTS

The point prevalence of Type II Diabetes Mellitus among adult population in Medicine Department of Himal Hospital Private Limited is 46 (23.93%) (0.23) (C.I= 0.20-0.26). Among 192 participants, there were 86 male and 106 female; DM was more prevalent among female (27) than male (19).

**Figure 1:**
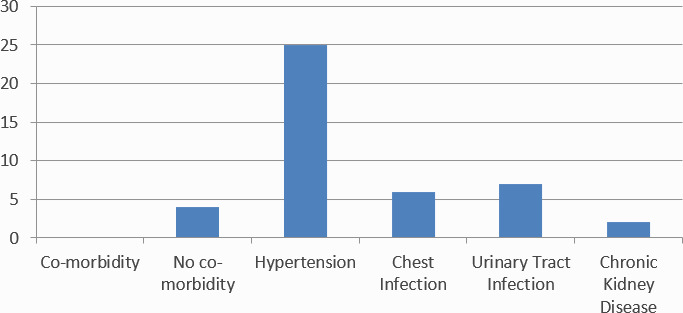
Age-wise Proportion of Type II DM.

Type II DM was found to be more prevalent 14 (30.43%) among the age group 70-80 followed by age group 60-70 and 80 plus (Figure 1).

**Figure 2:**
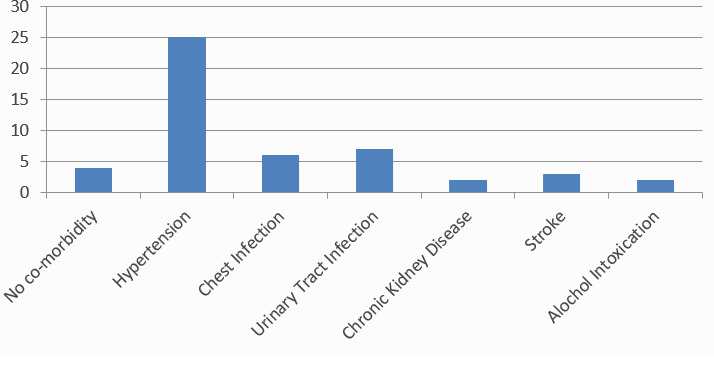
Co-morbidities associated with Type II Diabetic Cases.

Among the diabetic cases, the most common co-morbidity associated with Type II DM was hypertension 25 (54.34%) followed by UTI 7 (15.21%) (Figure 2).

Out of the total diabetic cases, only 8 (17.39%) presented with the complication of Diabetic ketoacidosis.

## DISCUSSION

Our study concluded the prevalence findings of Type II Diabetes Mellitus as 23.93% which was found to be higher than the similar studies with similar settings. This can be attributed to the increased risk factors secondary to urbanization and modern lifestyles.^4^

The systematic review done by Jayawardena et al reported the prevalence ratio of pre-diabetes and diabetes in Nepal as 19.5%;9.5% (2007;Urban).^7^ This finding was slightly lower than our study; however highlighted the rising epidemic of diabetes and the need for urgent preventive and curative strategies.

In our study, it was found that the diabetes prevalence increases as the age rises especially after age sixty. Similarly, the study done by Chhetri et al found that 25.9% of people were suffering from diabetes aged 60 years and older.^5^ This study highlighted that elderly populations are susceptible to many non-communicable diseases, including diabetes. This study also reported increased association of hypertension with diabetes. In our study, similarly, hypertension was the most commonly associated co-morbidity.

People with diabetes have a two-to three-fold increased risk of heart attacks and strokes. Our study also shows the association of DM with hypertension and stroke.^8^ Similarly, diabetes is also among the leading causes of kidney failure and is associated with Chronic Kidney Disease.^9^

The study done by Sah and Jha in a tertiary care hospital showed rising prevalence of diabetes with increasing age with more cases among male than female.^10^ In contradiction, our research shows more diabetic cases among female than male. This can be attributed to the different study settings and socio-demographic pattern.

The limitation of our study is that this study is limited to only one tertiary care centre, so the results of this study may not be generalizable to other place.

The cross-sectional nature of the study, does not allow the establishment of causality. Some of the cases of Diabetes may have missed which would affect the overall validity of the study. Quality of the documentation could not be as per the standard. It could be very time-consuming. The utility to using the clinical record audit to determine causation for patient outcome is limited

## CONCLUSIONS

The prevalence of Type II Diabetes Mellitus was found to be higher than the previous study done in similar settings. For better research findings in the future, the study involving large area with increased number of sample size is recommended.
